# Comment on Huțanu et al. Low Serum Vitamin D in COVID-19 Patients Is Not Related to Inflammatory Markers and Patients’ Outcomes—A Single-Center Experience and a Brief Review of the Literature. *Nutrients* 2022, *14*, 1998

**DOI:** 10.3390/nu14163387

**Published:** 2022-08-18

**Authors:** Patrick Chambers

**Affiliations:** Department of Pathology, Torrance Memorial Medical Center, Torrance, CA 90505, USA; pwc@gte.net

The article by Huțanu et al. [1] attempts to evaluate the protective effects, or lack thereof, provided by serum vitamin D against COVID-19.

Unfortunately, a serum level less than 20 ng/mL was selected to represent deficiency. The median for the deficient COVID-19 group was 11.85 ng/mL, while that for the “sufficient” COVID-19 group was 28.25 ng/mL. The median values for the four age groups amongst the healthy controls varied from 25–31 ng/mL.

The RDA of 600–800 IUs per day recommended by the National Academy of Sciences Institute of Medicine (IOM) was proven to be off by an order of magnitude [2]. This huge error was confirmed by several Canadian and American university research teams. The formal article was published in this very journal [2].

The IOM’s RDA for vitamin D still stands at 600 IUs to 800 IUs, but their Estimated Average Requirement (EAR) was subsequently raised from 20 ng/mL to 30 ng/mL.

An increased intake of an order of magnitude, e.g., 8000 IUs of D3 (cholecalciferol), would correlate with a serum level of at least 50 ng/mL. Dr. Fauci takes 6000 IUs D3 per day and some vitamin C.

Basically, all participants in both the healthy group and the COVID-19 group were vitamin D deficient. The study is statistically sophisticated, but the comparison seems meaningless.

The selected value of 20 ng/mL is woefully inadequate. Even 30 ng/mL falls well short [3].

Furthermore, vitamin D deficiency is often accompanied by magnesium deficiency. Mg^++^ is a required cofactor for:

1. The synthesis of the active form of vitamin D (three different enzymes) (Figure 1);
2. The synthesis of 7-dehydrocholesterol, the substrate for UVB synthesis of D3 (Figure 2);
3. The transport of D3 via VDBP (Figure 1);
4. The synthesis of PTH (Figure 1).

Any study on the efficacy of vitamin D would be immeasurably more meaningful if the results were adjusted for magnesium deficiency. Intracellular Mg^++^ (the only active form) is difficult and somewhat expensive to measure, but a simple survey of the study groups about symptoms of magnesium deficiency might be a step in the right direction.

## Figures and Tables

**Figure 1:**
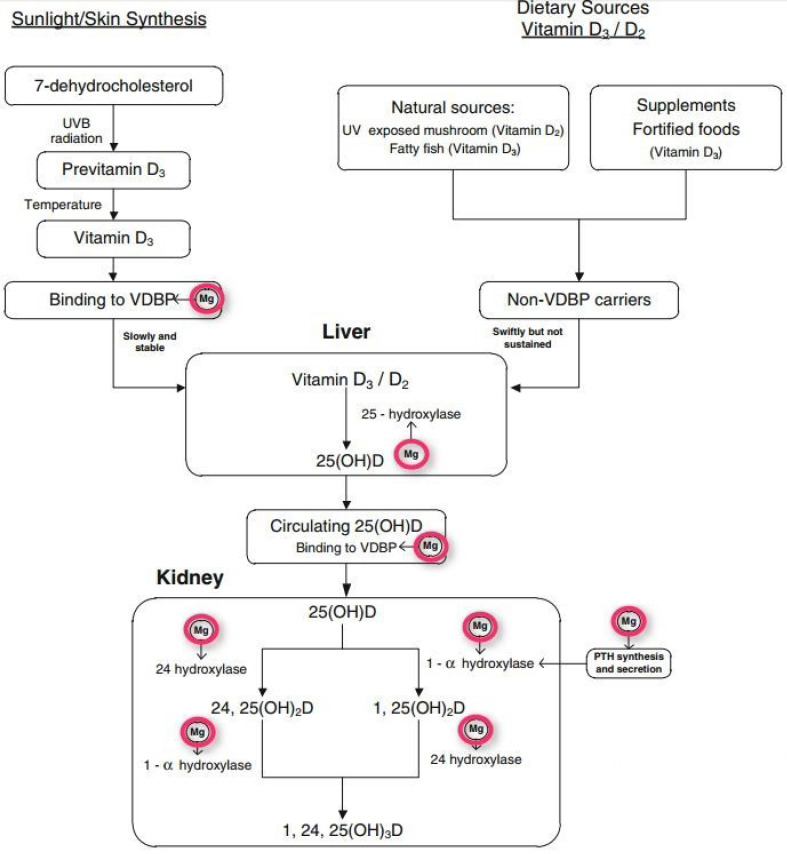
Magnesium is required for the synthesis of the active form of vitamin D and PTH.

**Figure 2:**
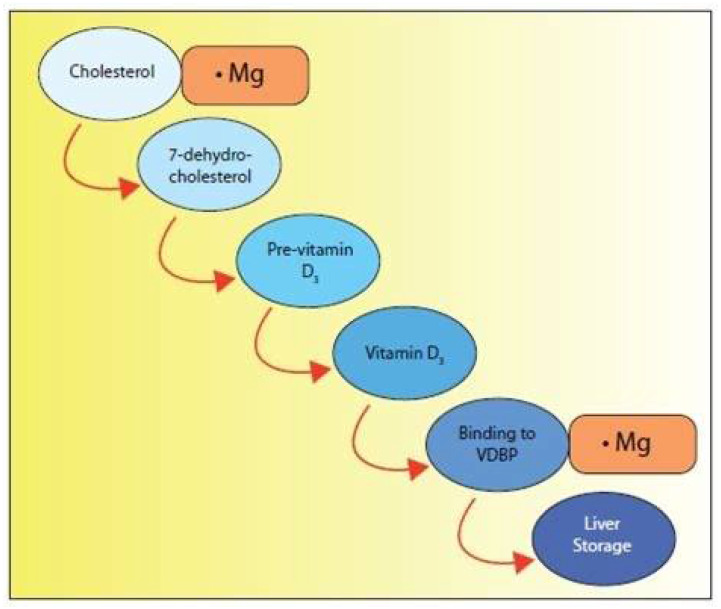
Even the synthesis of the substrate upon which solar derived D3 is produced requires magnesium.
